# Sensor Drift Compensation Based on the Improved LSTM and SVM Multi-Class Ensemble Learning Models

**DOI:** 10.3390/s19183844

**Published:** 2019-09-05

**Authors:** Xia Zhao, Pengfei Li, Kaitai Xiao, Xiangning Meng, Lu Han, Chongchong Yu

**Affiliations:** 1 Beijing Key Laboratory of Big Data Technology for Food Safety, Beijing Technology and Business University, Beijing 100048, China; 2 Shenyang Research Institute of China Coal Technology and Engineering Group, Fushun 113122, China; 3 State Key Laboratory of Coal Mine Safety Technology, Shenyang Branch of China Coal Research Institute, Shenyang 110016, China

**Keywords:** drift compensation, LSTM, SVM, gas recognition, the multi-classification ensemble learning model

## Abstract

Drift is an important issue that impairs the reliability of sensors, especially in gas sensors. The conventional method usually adopts the reference gas to compensate for the drift. However, its classification accuracy is not high. We propose a supervised learning algorithm that is based on multi-classifier integration for drift compensation in this paper, which incorporates drift compensation into the classification process, motivated by the fact that the goal of drift compensation is to improve the classification performance. In our method, with the obtained characteristics of sensors and the advantage of Support Vector Machine (SVM) in few-shot classification, the improved Long Shot Term Memory (LSTM) is integrated to build the multi-class classifier model. We tested the proposed approach on the publicly available time series dataset that was collected over three years by the metal-oxide gas sensors. The results clearly indicate the superiority of multiple classifier approach, which achieves higher classification accuracy as compared with different approaches during testing period with an ensemble of classifiers in the presence of sensor drift over time.

## 1. Introduction

In recent years, with the rapid development of the machine olfactory technology, the gas identification systems have been widely applied in many fields, such as food testing, medical diagnosis, and environmental monitoring [1,2,3]. In the gas identification systems, the gas sensors are often used as the core function for sensing, identifying, and measuring different gases. The key to the sensors is to realize the function of human smell to improve the accuracy of sensors [4,5]. However, the drift phenomenon is inevitable and it cannot be ignored during the use of the sensor over time [1]. There are several forms of the drifting, such as Zero and Span drift and Concept drift. Zero drift means that the reference deviates from a fixed value due to the influence of the external environment when the input signal of the amplifying circuit is zero. Span drift refers to a change of the coefficient and conversion factor of the value amplifier with the changes of time and temperature. Sensor drift implies the interference of some factors, such as the temperature of the surrounding environment, humidity, pressure, as well as the aging and poisoning effects of the sensor material (including external pollution, irreversible combination), which results in the sensor input signal that is involved in the interference signals. The external environment makes the interference signal continuously increase, which results in a gradual decline in data quality and the acquisition accuracy. The difference from the true value increases, and it is difficult to judge the type of gas based on the output value. Concept drift means that the data distribution recorded by the sensor has changed in machine learning. There are two main reasons for Concept drift [6,7,8]. First, the change in the sensor film microstructure that is caused by chemical and physical interaction is called ‘the real drift’ (for example, aging and poisoning leading to irreversible combination); second, changes in the operating system and the effect that is brought by external environment can be called ‘the second-order drift’ (such as temperature, the recording protocol, and the system lag). Various concept drift algorithms are classified according to the types and occurrence of drift. Concept drift can be compensated by using machine learning techniques.

Research show that, with time changing, the issue that sensor would drift is unavoidable. When the sensor is drifting, its performance is degraded, leading to the inaccuracy of the collected data. Therefore, we need to take some measures to compensate for it. Through the compensation technology, the classification of the gas can still be accurately identified in the case where the collected data is inaccurate. In the practical applications, the compensation methods of the sensor drift are divided into two categories, namely the hardware compensation and the software compensation. The hardware compensation refers periodical adjustments or regulations to the sensors, such as removing any poisoned and aged modules from the sensor’s surface film. The compensation renews the sensor and designs the system more effectively. However, hardware compensation cost a lot [7,8,9]. The software compensation includes the univariate method, the multivariate method, and the statistical machine learning method. In the univariate method, each sensor signal is independently calibrated and tends to compensate for a single sensor response, regardless of other sensors. The univariate method includes frequency analysis, baseline operation, differential measurements, and baseline operation. The univariate method has advantages, such as easy implementation and high time efficiency, so it has been widely used in practice. However, its disadvantage is that it is sensitive to the changes of the sampling rate [10,11]. The multivariate method tends to compensate for the entire sensor and further responses to the signal correction. When compared with the univariate method, the multivariate method uses information from the multiple sensors to simulate the drift, so that they can capture the complex drift effects. One of the disadvantages of the multivariate method is that it requires a frequent sampling and recalibration, which is laborious and expensive. The statistical machine learning method includes support vector machine and random forests.

Based on the traditional statistical machine learning methods, the multivariate data analysis reduces the dimension of data with features. Tom Artursson et al. have proposed a simple drift reaction method that is based on the Principal Components Analysis (PCA) and Partial Least Squares regression (PLS). The basic idea is to remove the components of the drift direction from the measurements [12], so that the sensor responding normalization classifier can be directly applied to the generated fixed data. M. Padilla et al. have proposed using signal processing techniques to achieve drift counteraction and also have supported to correct drift compensation in chemical sensor arrays by orthogonal signal correction [13]. Qi Liuhe et al. have mentioned that these techniques are on the basis of the linear hypothesis drift model and it has not been proven [14]. In addition, the implementation of these technologies requires a chemically stable reference gas that is highly correlated with the target gas as time passing in terms of sensor behaviors, which is difficult to apply in practice. When there is no explicit description for the sensor drift, the machine learning is the candidate for compensation the drift, which includes artificial neural networks, K Nearest Neighbor (KNN), Random Forest (RF), Support Vector Machine (SVM), and etc. Some deep learning models are gradually applied to classification studies [15,16,17]. Saswati Adhikari et al. have proposed an Artificial Neural Network (ANN) and KNN multi-classifier approach to improve the performance of classifiers and to mitigate the sensor drift [18]. Zhang et al. have proposed a domain adaptive Extreme Learning Machine (ELM) with the capability of handling drifting [19]. Liu et al. have combined the Geodesic Flow Kernel (GFK) and the popular regularization method to compensate for the gas sensor drift [20]. In recent years, with deep learning as a new research direction in the field of machine learning, Shen and others have utilized the Recurrent Neural Network (RNN) to capture the timing signals, which has predicted the sensor drift and it has reduced the number of sensor calibrations [21]. Long Short-Term Memory (LSTM) compensates for some issues, including the gradient disappearance and gradient explosion of RNN and the lack of long-term memory ability, which enables RNN to effectively utilize the long-range timing information. Wang et al. have proposed an LSTM prediction mode parameter optimization algorithm that is based on the multi-layer grid search, which has relatively strong applicability and a relatively high accuracy in the predictive analysis [22]. However, as time goes by, the quality of data collected by the sensor decreases. In the case that cross entropy is utilized as loss function for few amounts of data, the output of softmax in the LSTM model could lead to over-fitting. Moreover, the confidence range and threshold could not be practically determined.

Multi-classifier ensemble learning combines with a variety of learning algorithms, so the corresponding hypothesis space can be expanded, which reduces the drawbacks of a single learning algorithm [23]. With the efficacy of multi-classifier ensemble learning, this paper proposes a supervised learning algorithm that is based on a multi-classifier ensemble learning. With the objective to improve the calculation accuracy, we have developed a multi-classifier integrated with a new loss function and SVM for the base classifier LSTM, which greatly combines the advantages of SVM for small samples with the advantages of LSTM in time series. This multi-classifier can be integrated by the voting strategy with the normalized weighting. We select the ‘Gas Sensor Array Drift Dataset’ Dataset, which is a benchmark dataset available online at UCI machine learning repository, to verify the method. The proposed method is capable of compensation drift in gas sensors and it does not system re-calibration or background information, which makes it feasible for use in real time applications.

The rest of this article is organized, as follows. Section 2 is Data Processing. Section 3 describes the entire flow of the sensor drift. Section 4 consists of the analysis and results, and finally conclusions will be drawn in Section 5.

## 2. Data Processing

The data that were collected by the sensor have a high dimension and the overall processing amount is large. If the sensor drift compensation is directly performed on the original data, it is difficult to achieve the desirable effects. While considering this case, Vergara at al. firstly selected the features of origin data when creating the dataset [7]. The method in this paper is to do a correlational analysis in the selected dataset. Finally, the dataset is processed based on the correlational analysis coefficient, and the dataset will then be done in a normalization process. 

### 2.1. Data Acquisition

The dataset that was used in this study is collected in a controlled laboratory setup while using an array of sixteen metal oxide gas sensors that were manufactured by Figaro Inc. [24]. As for the preparation of this paper, it is a necessity that the sensor array consists of 16 pieces of Figaro commercial gas sensor with different sensitivity, among which each kind of sensor is equipped with four pieces of sensors. Table 1 shows the detailed information of the sensor arrays.

**Figure 1:**
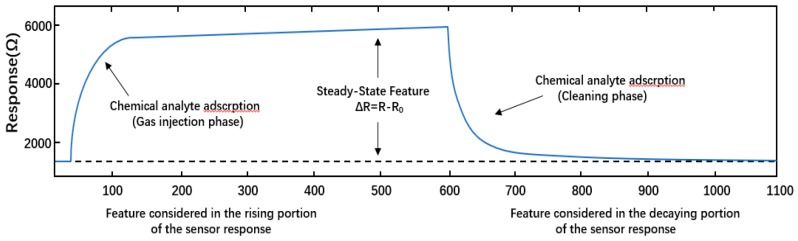
Typical information of sensor response. Typical response of a metal-oxide based chemical sensor to 30ppmv of Acetaldehyde. The curve shows the three phases of a measurement: baseline measurement (made with pure air), test gas measurement (when the chemical analyte is injected, in gas form, to the test chamber), and recovery phase (during which the sensor again is exposed to pure air; the recovery time is usually much longer that the gas injection phase).

Before this study, the needed datasets have been measured according to the next procedures. First, a constant flow of zero-stage dry air circulates through the sensing chamber, while the gas sensor array remains at a stable operating temperature (400 °C). This step measures the baseline steady-state sensor responses (the responses of the sensors in the absence of chemistries). The desired odorant concentration is then injected into the sensing chamber by a continuous flow system. Finally, in the third step (cleaning phase), the steam is evacuated from the sensor arrays and the test chamber is cleaned with the dry air before the newly measured concentration phase. The acquisition time for these measurements takes at least 300 s, including 100 s of a gas injection and at least 200 s of a recovery (cleaning). For purposes of processing, we consider the entire sensor responses after subtracting the baseline from each record. The sampling rate is set to 100 Hz. Finally, the measurement process that is described herein can be replicated for the subsequent measurements. The completed experimental setup of data acquisition process in given in [7]. Figure 1 shows typical information about the sensor response.

### 2.2. Feature Extraction

**Figure 2:**
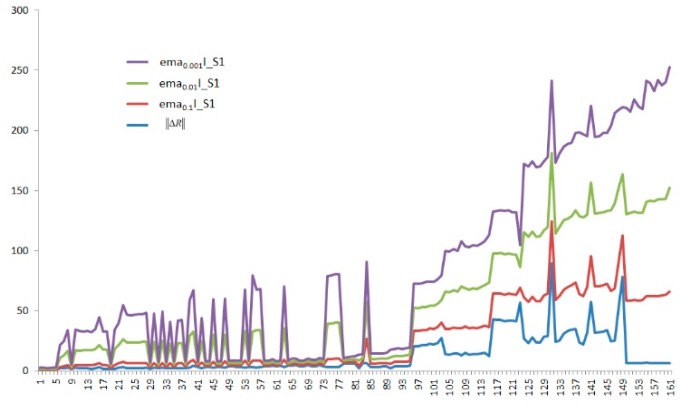
The results of the extraction feature.

Feature extraction is extremely significant in every chemo-sensory application [24], which could be described as a reflection of the sensor response under the lower-dimensional space, which preserves the most meaningful portion of the information that can be contained in the original sensor signals. Vergara et al. have considered two distinct types of features that exploit the whole dynamic process that occur at the sensor surface, including the ones that reflect its adsorption, desorption, and steady-state (or final) response of the sensor element [7]. The extracted features reflect transient response (desorption, adsorption) and the steady state response of the sensors [6]. The extracted steady state and transient features are computed as:

(1) $$||\Delta R||=\frac{\underset{k}{\max}r[k]-\underset{k}{\min}r[k]}{\underset{k}{\min}r[k]}$$

(2) $$\Delta R=\underset{k}{\max}r[k]-\underset{k}{\min}r[k]$$

where r[k] is the time curve of the sensor resistance and ΔR is the difference between the maximal resistance and the baseline. ||ΔR|| is the ratio of the maximal resistance and the baseline values; k is the discrete time indexing the recording interval [0,L] when the chemical vapor is present in the test chamber. The aggregate of features reflecting rising/decaying sensor response is evaluated by exponential moving average $ema_{a}$. The value of $ema_{a}$ is determined by calculating maximum/minimum y[k] for rising/decaying evaluation, respectively [25]. y[k] is calculated by the following formula:

(3) y[k]=(1−a)y[k−1]+a(r[k]−r[k−1])

where *k* is the discrete time indexing the recoding interval [0,L] when the chemical vapor is present in the test chamber, and *k* = 1,2, …, L. y[k] represents the real scalar, its initial state is set to zero, and the scalar a(a∈{0,1}) represents the operator’s smoothing parameter, namely f(a(r[i])), which refers to the quality of its feature and its time sequences [9]. Setting the three different values with 0.1, 0.01, and 0.001 of a to calculate the response with increase (I) and decay (D) in the sensors. The results that were obtained with the Equation (1) for the three values of a and the result of the extraction feature with Equation (1), as shown in Figure 2.

For each gas sensor in the array, two steady and six transient features are computed and a feature vector of 128 (16 sensors × 8 features) features is recorded. The order in which the proposed features are placed in the feature vector is shown in Table 2. S1 in the Table 2 denotes Sensor 1 (S1); S2 represents Sensor 2 (S2), and so on until the Sensor 16 (S16); $ema_{a}$ (a∈ {0.1, 0.01, 0.001}) represents the exponential moving average.

### 2.3. Correlation Analysis

Table 2 shows the results of correlation analysis of the dataset. According to the result that the correlation coefficient between the two variables is over 0.9, removing any variable and reducing the dimension of the data, which we could fully utilize the data in low dimension. The main methods of data correlation analysis could be divided into Pearson Product Moment Correlation Coefficient (PPMCC), Kendall rank correlation coefficient (Kendall), and spearman’s correlation coefficient for ranked data (spearman). These three methods all reflect the direction and extent of the trend between two variables. When compared with PPMCC, spearman could not perform well in accuracy and it is insensitive to data errors and the extreme value. Kendall is a rank correlation coefficient and it calculates the objects that are categorical variables, namely the classification categories of variables.

In conclusion, PPMCC is the most suitable for the evaluation of sensor drift data. Assuming $X_{i}$ for the *i*-th variables; $\rho_{x_{i},y_{i}}$ for the Pearson correlation coefficient between the *i*-th variables and the *j*-th, the Pearson correlation coefficient between two variables could be expressed as:

(4) $$\rho_{x_{i},y_{i}}=\frac{\mathrm{cov}(X_{i},X_{j})}{\sigma X_{i}\sigma X_{j}}=\frac{E[(X_{i}-\mu_{X_{i}})(X_{j}-\mu_{X_{j}})]}{\sigma_{X_{i}}\sigma_{X_{j}}}$$

where $X_{i}$ and $X_{j}$ are two eigenvalues of 128 features in Table 2. $\mathrm{cov}(X_{i},X_{j})$ is the covariance between the *i-*th variable and the *j-*th variable, $\sigma_{X_{i}}$ is the population standard deviation of the *i-*th variable, and $\mu_{X_{i}}$ refers the population mean difference of the *i-*th variable. Calculate the covariance and standard deviation of the datasets, and obtain the Pearson correlation coefficient of the sample as:

(5) $$g=\frac{\sum_{i=1}^{n}\left(X_{i}-{\bar{X}}_{i})(X_{j}-{\bar{X}}_{j}\right)}{\sqrt{\sum_{i=1}^{n}{\left(X_{i}-\bar{X}\right)}^{2}}\sqrt{\sum_{i=1}^{n}{\left(X_{j}-{\bar{X}}_{j}\right)}^{2}}}=\frac{1}{n-1}\sum_{i=1}^{n}\left(\frac{X_{i}-\bar{X_{i}}}{\sigma X_{i}})(\frac{X_{j}-\bar{X_{j}}}{\sigma X_{j}}\right)$$

where $\frac{X_{i}-\bar{X_{i}}}{\sigma X_{i}}$ indicates the standard score for the $X_{i}$ sample; ${\bar{X}}_{i}$ is the average of the samples; $\sigma X_{i}$ is the standard deviation of the samples; g is described as the Pearson correlation coefficient; and, *n* refers to the sample size. The range of g is between [–1,1] [26]. If g > 0, it means that the two variables are positively correlated. There remains other condition, such as g = 0, signifying that there is no linear correlation between the two variables and g < 0, showing that the two variables are negatively correlated. Correlation analysis is performed on the entire dataset, and one of the variables with g > 0.9 is removed from the variable and only one of the variables is reserved as a processed dataset. Table 3 shows partial results of data correlation processing, where null represents the deletion of data from the original location.

## 3. Improved LSTM and SVM

### 3.1. Sensor Drift Model Based on Ensemble Learning

**Figure 3:**
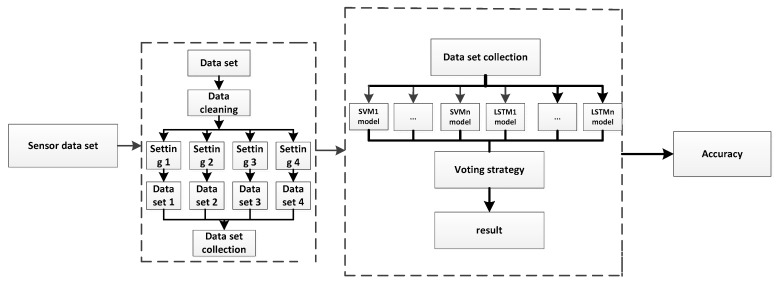
Improved Long Short-Term Memory (LSTM) and Support Vector Machine (SVM) (ILS) Flow Chart.

The dynamic behavior of the sensor drift cannot be calibrated. The machine learning methods make the adaptive sensor drifts of the model more attractive. The proposed method is based on the combination with the SVM and the improved LSTM to achieve precise gas classification in any concentration. The proposed Improved LSTM and SVM (ILS) mainly comprise three folds, namely the processing of data, the integration of an ILS model classifier, and the model evaluation of the model, as shown in Figure 3.

The steps are implemented as follows.

1. The dataset is purified to eliminate the noisy data and then the datasets are processed through four settings. Moreover, four different kind of datasets are obtained under the four settings. Datasets are numbered in turn, forming Dataset 1, Dataset 2, Dataset 3, and Dataset 4. In the following experiments, the four settings are explained. Moreover, setting 3 and 4 are processed while using correlation analyses.
2. Dataset 1, Dataset 2, Dataset 3, and Dataset 4 are used as inputs to the improved LSTM and SVM models, respectively, and then eight independent classifiers (there are four improved LSTM classifiers and four SVM classifiers) are trained. In the case of the same test samples, the eight classifiers output different classification results, which are used to obtain the final predicted outcomes by the normalization and weighted voting strategy.
3. Classification accuracy rate is adopted to evaluate the classifier performance of the ILS model.

**Figure 4:**
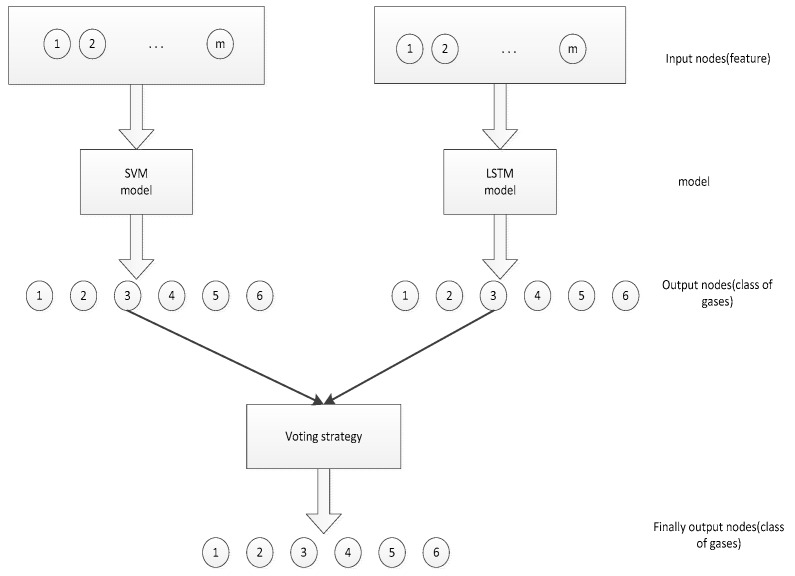
Input and output of two classifiers.

The input and output of the two types classifiers are shown in the Figure 4. The input is the characteristic of each dataset, and the output layer is allocated with six different gases. Finally, the classifier output six different gases through the voting strategy. 

We use an ensemble of classifiers to detect and cope with the sensor drift. A set of features x as input and a class label (a gas/analyte in our problem) y as output. In every dataset *t*, we use a batch of $S_{t}=\{(x_{1},y_{1}),\ldots ,(x_{n},y_{n})\}$, and we also train the classifier. A simple and intuitive way is to assign weights to classifiers according to their prediction performance on batch $S_{i}$. We trained an ensemble of multi-class classifiers using the method in Algorithm 1. The Algorithm 1 is given below.

**Algorithm 1**. Algorithm to cope with concept driftInput: Dataset $Y=\{y_{1},\ldots ,y_{n}\}$ and $X=\{x_{1},\ldots ,x_{n}\}$ Output: final classifier: weights and classifiers Method: ILS 1: for *t* = 1, …, N do 2:  Receive $S_{t}=\{(x_{1},y_{1}),\ldots ,(x_{n},y_{n})\}$ 3:  Train a classifier (SVM) on $S_{t}$ 4:  Estimate the weight $\left\{\phi_{1},\ldots ,\phi_{t}\right\}$ by the techniques described in the text 5: end for 6: for *t* = 1, …, N do 7:  Receive $S_{t}=\{(x_{1},y_{1}),\ldots ,(x_{n},y_{n})\}$ 8:  Train a classifier (LSTM) on $S_{t}$ 9:  Estimate the weight$\left\{\eta_{1},\ldots ,\eta_{t}\right\}$ by the techniques described in the text 10: end for

We can assign weights according to the prediction performance of classifiers on the most recent batches. In addition, we can simply estimate a single set of weights $\left\{\phi_{1},\ldots ,\phi_{t}\right\}$ and $\left\{\eta_{1},\ldots ,\eta_{t}\right\}$ by using the multi-class classifier prediction accuracies on every batch. The predications are based on the improved voting, as below.

#### 3.1.1. SVM Base Classifier

ILS model uses the SVM classifier as a base classifier in this paper. When dealing with linearly indivisible samples, SVM transforms themselves from low dimension feature space to high dimension by the non-linear mapping method, SVM constructs the optimal hyperplane in high dimensional space by using different kernel functions to make them linearly separable. It proves that a plurality of classification sets combined with LSTM and SVM is better than a single classifier model.

The learning method of LSTM is based on the principle of experience minimization. When the number of training sample is large enough, this method can provide the good compensation effects in the sensor drift, but it usually causes overfitting if the training sample is not enough. With regard to this defect, the principle of structural risk minimization can improve it, this principle can greatly reduce the generalization errors in the training set data, weaken the complexity of machine learning, and control the predicted risks of the entire sample set while ensuring classification accuracy. SVM is on the basis of this principle, and this principle of structural risk minimization can be described as:

(6) $$L=\frac{1}{2}{\left\|w\right\|}^{2}-\sum_{1}^{m}\alpha_{i}(y^{i}(w^{T}x^{i}+b)-1)$$

where ‖w‖ is the two-norm of the vector; $\frac{1}{2}{\left\|w\right\|}^{2}$ can be expressed as $\frac{1}{2}\sum_{1}^{m}w_{i}^{2}$, namely the L-2 regularization term. The second term in the above formula (6) represents the empirical risk. Where x represents the input characteristics of the training sample and y represents the output of the training sample set. $w=(w_{1},w_{2},\ldots ,w_{m})$ is the normal vector. T represents the transpose. Additionally, b is the offset, which determines the distance between the hyperplane and the origin. m is the number of input sample instances. The principle of minimization of structural risk minimizes the generalization errors of the training set data, and it reduces the complexity of the learning machine while ensuring the classification accuracy, so that the expected risk on the entire sample sets could be controlled. Finally, the SVM base classifier has been selected in this paper.

#### 3.1.2. Improved Base Classifier of LSTM

LSTM is a special form of RNN. Among many RNN variants, the LSTM model compensates for the gradient disappearance and explosion of RNN and the lack of a long-term memory capacity, which enables LSTM to effectively utilize long-range timing information [22,27]. Generally, this model is divided into three levels: the input layer, the hidden layer as well as the output layer. Given sequence $X=(x_{1},x_{2},\ldots ,x_{n})$ can calculate the hidden layer sequence $H=(h_{1},h_{2},\ldots ,h_{n})$ and the output layer sequence $Y=(y_{1},y_{2},\ldots ,y_{n})$ could be obtained by the iterative equations from the 7th to 9th.

**Figure 5:**
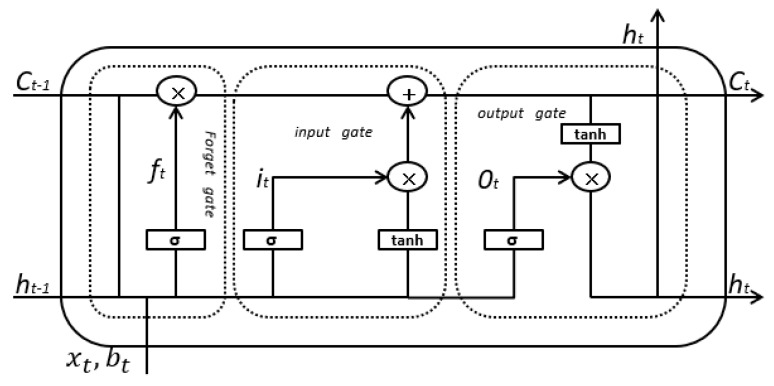
LSTM Unit Structure.

The forward calculation method of LSTM model cell can be expressed as:

(7) $$f_{t}=\sigma (w_{f}\cdot h_{t-1}+w_{f}\cdot x_{t}+b_{f})$$

(8) $$i_{t}=\sigma (W_{i}\cdot [h_{t-1},x_{t}]+b_{i})$$

(9) $$o_{t}=\sigma (w_{o}\cdot h_{t-1}+w_{o}\cdot x_{t}+b_{o})$$

(10) $${\tilde{C}}_{t}=\tanh(W_{C}\cdot [h_{t-1},x_{t}]+b_{C})$$

(11) $$C_{t}=f_{t}*C_{t-1}+i_{t}*{\tilde{C}}_{t}$$

(12) $$h_{t}=o_{t}*\tanh(C_{t})$$

where $f_{t}$, $i_{t}$, and $o_{t}$ are, respectively, the results of the forgotten gate, the input gate, and the output gate state settlement result; $W_{f}$, $W_{i}$, and $W_{o}$ are, respectively, weight matrix of the forgetting gate, the input gate, and the output gate; $b_{f}$, $b_{i}$, and $b_{o}$ are, respectively, bias terms of the forgetting gate, the input gate, and the output gate; $h_{t}$ is the final output of current neurons; $C_{t}$ is the unit state input at time *t* + 1; $W_{c}$ is the weight matrix in a unit state; and, $b_{c}$ is the bias of the input unit status. σ and X are sigmoid and hyperbolic tangent activation functions, respectively. According to the calculation between the information of the previous forgotten state and the current state information input gate, Figure 5 shows the cell unit structure of the LSTM model.

The LSTM backpropagation is similar to the Back Propagation Through Time (BPTT) algorithm. The steps of the LSTM model are as follows:

1. calculate the hidden layer. Output the upper and lower boundaries of the connection weights of the neurons and initialize the weights and thresholds;
2. calculate the output value and the error function value;
3. update the weight and threshold of the output neuron;
4. calculate the error value of the hidden layer neurons and update the weights and thresholds; and,
5. repeat it from step 2 to step 4 until the training model converges or reaches the number of training sessions.

**Figure 6:**
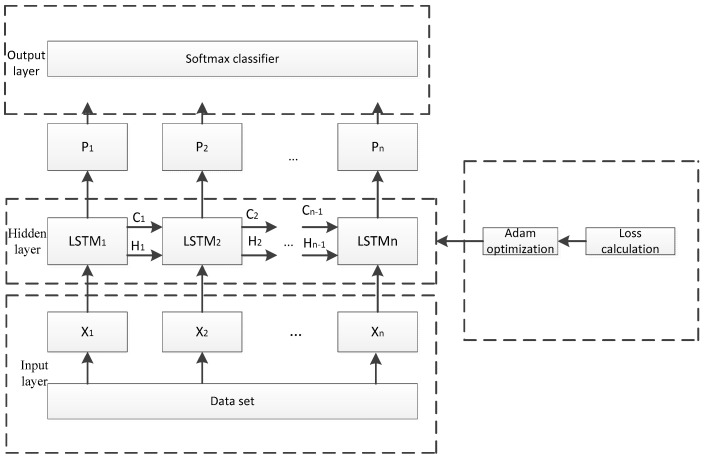
The Framework of The LSTM Time Series.

The overall framework of the LSTM model that was constructed in this paper is shown in Figure 6, which includes three functional modules: the input layer, the hidden layer, and the output layer. The input layer is responsible to process data in the dataset to meet the network input requirements. The LSTM cells that are shown in Figure 5 were used to construct a single-layer circulatory neural network in the hidden layer, which adopts the Adam optimization. The output layer can classify the results of LSTM training, further analyzing its accuracy. 

X as the input of the hidden layer, is then calculated in each single cell, respectively, with the forward propagation for training X and back propagation for optimizing loss function with Adam Optimizer. LSTM model outputs to $h_{i}$, where $C_{n}$ and $H_{n}$ represent the state and output of the previous LSTM cell, respectively. We choose cross entropy as the basis for the calculation formula:

(13) M(p,q)=−∑q(x)logp(x)

where p(x) is the distribution of predictions and q(x) is the distribution of the dataset. The SoftMax output is employed in this study. If cross entropy is directly chosen as the loss function, it could simulate the max operation well for the fact that exp function in Softmax is monotonically increasing, which assigns a high value to a node and low value to the remaining nodes to a. Additionally, it polarizes the results, which would lead to the weakened ability of error correction in practical application. With the increase of the experimental batch, the interference signals in the data that were collected by the sensor gradually increase and even dominate the main signals, which requires a reduction on the max operation to weaken the influence of such signals on the results and improve the accuracy of the model. More importantly, it is difficult to determine a confidence interval and set the threshold in practice. Therefore, it is necessary to improve the loss function. The improved loss function will be shown in the next section.

There are many types of gradient-based optimization algorithms, such as stochastic gradient descent, Stochastic Gradient Descent (SGD), and Root Mean Square Prop (RMSProp). This paper adopts Adaptive Moment estimation (Adam). The Adam algorithm integrates the advantages of the AdaGrad with RMSProp algorithms when compared with other optimization algorithms. In the process of parameter updating, firstly calculate the first and second moments, and then revise their deviation. After that, adopt the modified versions to sum the updated parameters and finally update the new version by the updated parameters. The Adam algorithm performs best in the practical application when compared with other stochastic optimization methods. 

#### 3.1.3. Multiple Classifiers Strategy

The multi-classifier combination is to make every classifier solve the same original tasks and combine the results of each model by a specific voting strategy to obtain a better global model.

This paper conducts a theoretical analysis of ensemble learning, while considering the six-classification problems y∈{1,2,3,4,5,6} and function f, while assuming that the error rate of the individual classifier is δ, namely a formula of Individual classifier $h_{i}$:

(14) $$M(h_{i}(x)\neq f(x))=\delta$$

where $h_{i}(x)$ indicates the predicted results and f(x) represents the real results if the integration is assembled with P individual classifiers by the simple voting. If more than half of the results of individual classifier are correct, the final determined is on the right,

(15) $$N(x)=sign(\sum_{i=1}^{P}h_{i}(x))$$

If the error rate of the classifier is independent of each other, the Hoeffding inequality shows that the ensemble error rate is

(16) $$M(H(x)\neq f(x))=\sum_{k=0}^{\left|P/2\right|}\left(\begin{array}{c} P\\ K \end{array}){\left(1-\delta \right)}^{k}\delta^{P-k}\leq \exp(-\frac{1}{2}P{\left(1-2\delta \right)}^{2}\right)$$

P is showed at the above Equation (16). It shows that the ensemble error rate will exponentially decline with the increase of the number of individual classifiers increase in the integration. However, in the practical experiments, it has been found that the error rates between individual classifiers are not independent, so the key of ensemble learning is the way of combination between individual classifiers. Although there exists the simple voting method, it is still difficult to achieve the impacts that effectively improves the accuracy of results in six-classifications. Therefore, we propose a normalized voting strategy to classify more than one kind of situations, which is to say, using a weighted sum of the weights to calculate the maximum output as a final result. Next, the multiple classification vote strategy will be illustrated in detail:

(17) $$y_{i}=\arg\max\sum_{i=1}^{n}\frac{\theta_{i}-\min}{\max-\min}f_{i}(x)$$

where $y_{i}$ represents the results predicted by the classifiers; x expresses the input; $\theta_{i}$ expresses the accuracy of the *i-*th classifier; $f_{i}(x)$ expresses the output of the *i-*th classifiers; and, min and max, respectively, indicate the minimum and maximum accuracy. Afterwards, normalize. The accuracies of all classifiers are then normalized because a normalized model can remove the model with the lowest accuracy. After that, use the weight of other remained models to get relatively high accuracy results.

### 3.2. Improved LSTM Model

#### 3.2.1. Improved Loss

The classification polarization led by the output of softmax function in the LSTM model may cause over-fitting. In practical, the confidence interval cannot be well determined and the threshold is hard to set. Therefore, we have developed a new *loss* function that is based on cross-entropy. The following *loss* function is designed:

(18) $$loss=\arg\min\sum_{i=1}^{m_{t}}\sum_{j=1}^{t}\max[-(1-\varepsilon )y_{i}\log(\beta_{j}f_{j}(x_{i})-\varepsilon /6y_{i}\log\beta_{j}f_{j}(x_{i}))-\varphi ,0]$$

where φ is the minimum value that can tolerate the error setting; ε is the threshold; $\beta_{j}f_{j}(x_{i})$ is the result of the forecast; and, $y_{i}$ is the data of real distribution. In this paper, the Figure 6 in denominator indicates that it uses the six classifications. The purpose is to fit the even distribution and reduce the overfitting. The importance of Max operator can be shown by an inequality, $-(1-\varepsilon )y_{i}\log(\beta_{j}f_{j}(x_{i})-\varepsilon /6y_{i}\log\beta_{j}f_{j}(x_{i}))\geq \varphi$. It can be implied that, whenever the loss is greater than φ, namely the difference between the predicted and the actual category is greater than the value φ, the loss returns to its configure. Otherwise, it will be the value zero. At the same time, regularization is introduced into the loss function to deal with the problem of sensor drift identification and correction by using the multi-classification model, so as to more accurately classify the sensor drift.

#### 3.2.2. Regularization

Complex neural network models are prone to be over-fitting. The regularization techniques are widely applied in machine learning. The function is to prevent the model from over-fitting and improve the generalization ability of models. The regularization method actually eliminates the singularity by separating the curves with different tangent lines at the singular points of the irreducible plane algebraic surface [28]. Add constraints to the minimization of the empirical error function, for example, L0 norm, L1 norm, and L2 norm. The constraint has a guiding function, and the fit in the optimization function tends to choose the direction of the constraints with the gradient descent, so that the final result tends to be constrained situation. L0 norm is:

(19) $${\left\|x\right\|}_{0}=\sum_{i=0}^{n}x_{i}^{0}$$

Using the L0 norm to regularize the parameter matrix is suitable for the feature matrix, but it is difficult to achieve optimization. The L1 norm is:

(20) $${\left\|x\right\|}_{1}=\sum_{i=0}^{n}\left|x_{i}\right|$$

Calculate the sum of the absolute values of the elements in the vectors, namely the L2. L2 norm is:

(21) $${\left\|x\right\|}_{2}=\sqrt{\sum_{i=0}^{n}{\left|x_{i}\right|}^{2}}$$

The regularization of L2 norm is to minimize the regularization term ‖w‖, which makes every element in W with a minimum, close to zero. However, it is different from L1 norm. Not every element is zero, but it is just close to zero.

**Figure 7:**
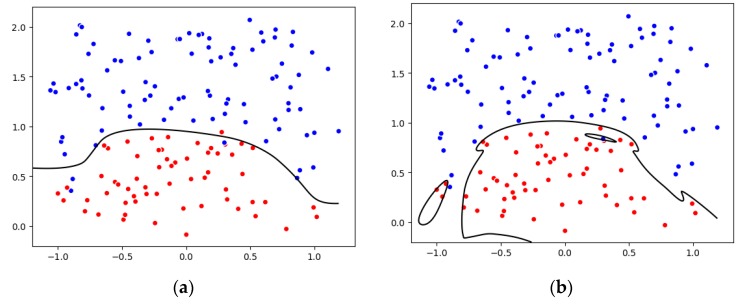
Synthesize two-dimensional data to illustrate regularization, (**a**) normal, (**b**) overfitting.

Figure 7 sets the L1 norm as an example of the regularization. We randomly generate two color points and divide both points into the two-dimensional space by a simple logistic regression. It can be seen that Figure 7b could better distinguish the points. However, when we add some extra data, and then the curve changes significantly. Thus, it is essential to achieve a more stable and generalized curve to prevent the over-fitting situation. As a result, our research team introduces regularization to enhance the stability and robustness of the model.

## 4. Analysis and Results

### 4.1. Experimental Datasets and Environment

The datasets that were adopted in this study come from ‘Gas Sensor Array Drift Dataset’ in the UCI machine learning repository. The primary purpose of making this dataset freely accessible on-line is to provide an extensive dataset to the sensor and artificial intelligence research communities to develop and test strategies to solve a wide variety of tasks, including sensor drift, classification, regression, among others. The datasets consist of 13,910 measurements of 16 chemical sensors from January 2008 to February 2011 (36 months). These sensors have been exposed to six different gases with different concentration levels. The resulting dataset includes the recordings of six different pure gaseous substances, such as Ammonia, Acetaldehyde, Acetone, Ethylene, Ethanol, and Toluene, respectively, dosed at a wide variety of concentration levels in the intervals (50,1000), (5500), (12,1000), (10,300), (10,600), as well as (10,100) PPMV [9]. The dataset is organized into 10 batches, with each batch containing diverse gas combinations shown in the tables below and the number of measurements per month, respectively, to handle the dataset conveniently. This reorganization of the data is to ensure that there is sufficient experimental data in each batch and the number of experiments is as evenly distributed as possible. The goal of this study is to distinguish among six different gases regardless of their concentration levels.

The labels in the tables indicate that the gas type 1 is ethanol; type 2 is ethylene; type 3 is ammonia; type 4 is acetaldehyde; type 5 is acetone; and, type 6 is toluene. Each of the possible gas type-concentration pairs has been sampled without any particular order. The resulting dataset consists of 13,910 recordings (time series sequences) collected more than 36 months [7,29]. Moreover, as observed in Table 4, the last batch containing 3600 measurements from the same analytes is purposely collected five months after the sensors are powered off. This five-month gap plays an important role in this paper not only because it allows us to validate our proposed method on the annotated set of measurements collected after five months, but because, during this time, the sensors are prompted to severe contamination, for it is easy to make external interferent irreversibly get attached to the sensing layer. Batch 10 as a test set can effectively validate the method that we use.

**Figure 8:**
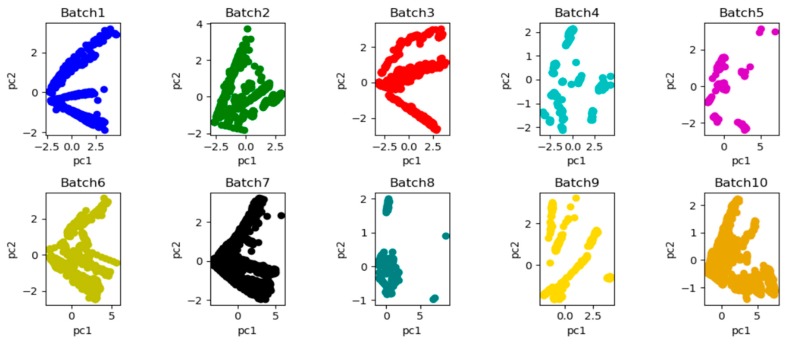
The performance of principal component analysis (PC1 vs. PC2) was performed on the original data of 10 batches by principal component analysis (PCA) (two-dimensional subspace distribution of 10 batches respectively), and the observation of the significant changes in data spatial distribution caused by drift could be observed.

Principal component analysis (PCA) [30] is carried out for these 10 datasets batches in order to intuitively observe and analyze the distribution of these 10 batches in the datasets. Figure 8 shows the influence of drift on data distribution. As time goes by, there is a significant bias in the two-dimensional subspace distribution between batch 1 and other batches because of the drift.

It is worth noting that the dynamic behavior of sensors after drift cannot be calibrated, and it is more valuable to use machine learning and data adaptive methods to compensate the sensor drift.

The computer setting in this experiment is as follows: a processor Intel Core i5-7300 HQ with 2.5 frequency GHz and the 3.5 GHz maximum frequency. The RAM is 8G. The operating system is windows 10 (6 4 bits) and programming language is python 3.5.2. The ensemble development environment is PyCharm 2017.1.2. The LSTM program model adopts the package in the TensorFlow 1.12.0 Python package.

### 4.2. Experimental Process and Results

#### 4.2.1. Drift Experiment

In the experiment, the SVM model and the kernel function that is [sigmoid, linear, poly] are selected, and the range of C, namely the penalty factor, is [$2^{-5},2^{-4},\ldots ,2^{9},2^{10}$]. According to the classification accuracy, we select the optimal kernel function and penalty factor. Eigenvalues in the training and testing sets are normalized into the range [−1,1]. If the testing operation is on batch 1, 1/5 of the batch 1 is set to be the test set and the remaining data is set to be training set. Subsequently, we verify the classification accuracy on the test set of batch 1. When batch 2–10 is used as the test set, batch 1 is used as the training set to train SVM classifier. According to the Table 5, the performance of the classifier would change with the experiment batch, namely the time changes (Table 4 shows data collection in batch 1–10 with time). The performance of the classifier would decrease, which can be an indicator of the sensor drift (that is to say, the lower rate the classification, the more severe the drift phenomenon). The experiment in this paper verifies that the data collected by the sensor is drifting, and the drift reduces the performance of the classifiers.

#### 4.2.2. Base Classifier

We consider the following four sets of data. According to the diverse setting of each dataset, these datasets are named as dataset1, dataset2, dataset3, and dataset4, respectively. The four settings are as follows:

- Setting 1: Use batch (*k* − 1) as the training set and batch *k* as the testing set, where *k* = 2, 3, 4, 5, 6, 7, 8, 9, 10.
- Setting 2: Train a multi-class classifier with data from only the previous month and test it on the current month.
- Setting 3: Use PPMCC to process the dataset, Afterwards, follow the Setting 1 for training and testing.
- Setting 4: Use PPMCC to process the dataset, Afterwards, follow the Setting 2 for training and testing.

In Setting 1, the latest batch is selected as the training set to reduce the differences between the test set and the training set due to the sensor drift. However, because of the different amount of data in different batches, the latest batch of the data is not various enough to involve the types of all data, so that we set all of the data before the latest batch as the training set in Setting 2. The SVM trained in Setting 2 is a strong baseline, because it sees the most recent batch of examples that is not corrupted by drifted data from the past, and data in setting 2 was not analyzed for correlation. Therefore, we use the classification accuracy of SVM in Setting 2 as an uncompensated comparison. Settings 3 and Setting 4 reduce the dimension of the two datasets formed by the Setting 1 and Setting 2 through PPMCC.

A. SVM model

The dataset is divided into 10 batches. The SVM model is utilized for four different kinds of datasets to compensate for the sensor drift. The SVM model chooses the kernel functions [ sigmoid, linear, poly]. The penalty factor C is selected from the range [$2^{-5},2^{-4},\ldots ,2^{9},2^{10}$]. Subsequently, we compare the classification accuracy rate and choose the optimal kernel function and penalty factors.

**Figure 9:**
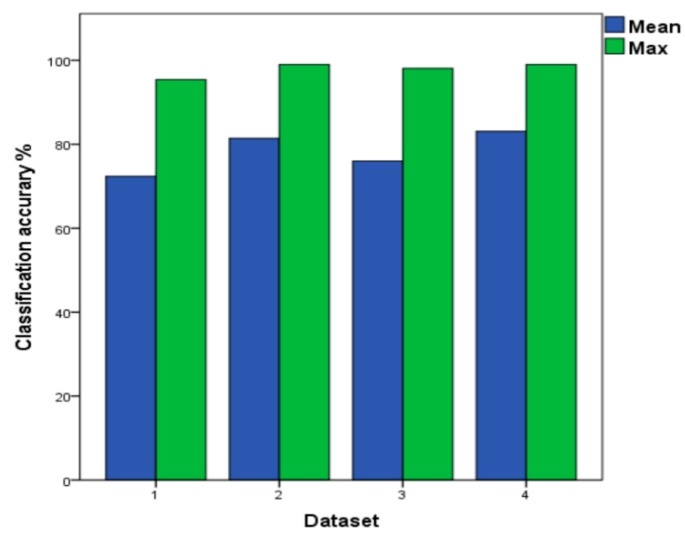
The Mean and Maximum Values of SVM under the Four Datasets. Green: the highest classification accuracy in every dataset; Blue: the average classification accuracy in every dataset.

Table 6 shows that the SVM model has the highest classification accuracy rate, reaching 99% in the test dataset included in batch 2–10. As shown in Figure 9, the SVM model is tested on four different datasets. It could be implied that the classification accuracy rate of the dataset 2 and dataset 4 are both up to 99%, and the average classification accuracy of dataset 4 is 83.1%. Under the four different datasets, the classifier using batch 10 as the testing set has the lowest classification accuracy of 34.5%. We believe that there is a six-month difference between the dataset batch 9 and batch 10, and a large number of interference signals appear in the data that were collected by sensors in batch 10, which results in a low accuracy of the classifier. Using batch 10 in the dataset 4 as a test set, the classification accuracy of batch 10 reaches 70.6%. The SVM trained is the baseline and the average classification accuracy is 81.4% in dataset 2 of the Table 6. When compared to the baseline, the compensation is improved by 1.7% in dataset 4. Due to the dataset 4 with the highest average classification accuracy, the results of dataset 4 are adopted to compare with our proposed method ILS to test different performance of models.

B. LSTM model

We apply four kinds of dataset described above as the input of LSTM model. Subsequently, we choose relu as the activation function in the LSTM model and set different rates of learning (learning_rate = 0.001, 0.0015, 0.0025, 0.005, 0.0075). The learning_rate with the highest test accuracy obtained by dynamic changes is regarded as the current learning_rate. Because of the number of training sets that are based on the dynamically increased dynamic setting, the number of iterations is set to 50 times of the number of rows in the current training set. LSTM model is divided into four layers. The input of the input layer comes from different datasets; the output layer distributes six different gases, and there are also two hidden layers. We choose cross-entropy as the loss function and use Adam as the optimizer.

**Figure 10:**
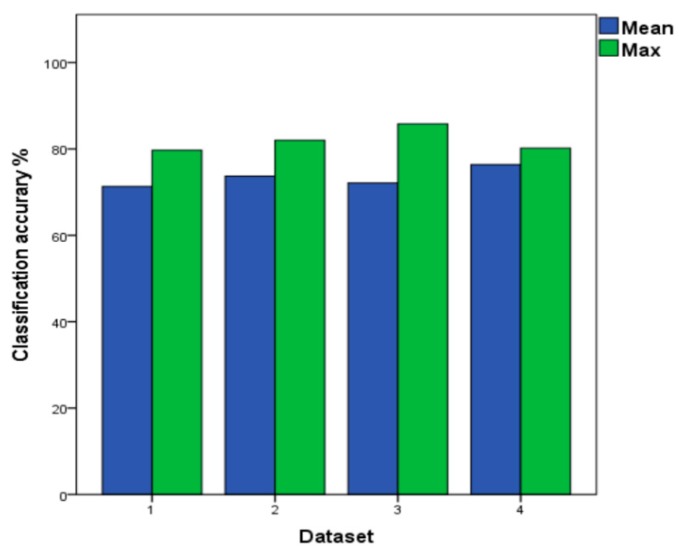
The Mean and Maximum Values of LSTM under the Four Datasets.

As shown in Table 7, when the LSTM model chooses batch 10 (six months out of batch 9) as the test data, the accuracy reaches 78.6%. When compared with a SVM model with the same data equipment, LSTM is more accurate, with a higher 5.8% accuracy. Thus, for the data with the predicted long-time differences, the LSTM model classifier performs better. As shown in Figure 10, the LSTM model as a test set is applied in the batch 2–10. The highest classification accuracy among the four different datasets is 85.7%, while SVM model’s accuracy is 99.0%. Hence, the SVM model is more suitable for the datasets that are small and whose time span is not too long. The average classification accuracy in the dataset 4 among four datasets is 76.4%, and the results of dataset 4 will be compared with our method that is proposed below.

C. Improved LSTM model

The same four datasets mentioned above are used as above for the improved LSTM model input. In this improved LSTM model, the loss function of the LSTM model is updated, as shown in equation 17. The rest module of the improved LSTM model is configured in the same way as the above LSTM model.

**Figure 11:**
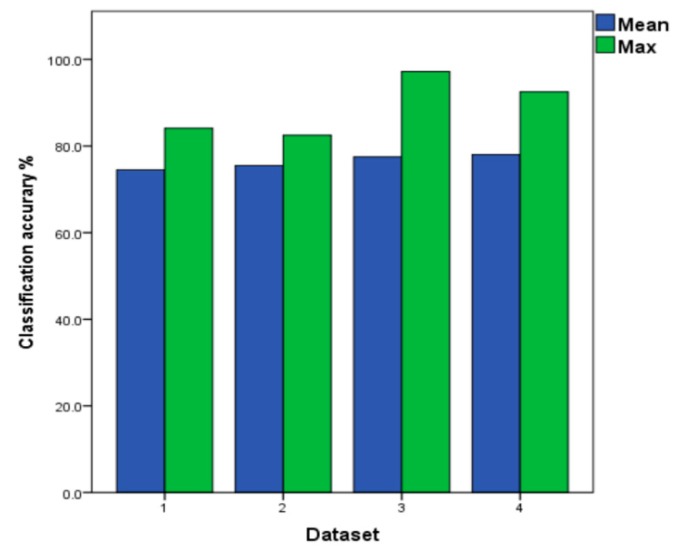
The Mean and Maximum values of Improved LSTM under the Four Datasets.

The improved LSTM shows a higher classification accuracy when batch 10 is the test set when compared with the LSTM (batch 9 differs from batch 10 by six months). In dataset 4 of the Table 8, the classification accuracy reaches 83.3%, increasing by 4.7%. It can be verified that the improved loss function is more adaptive to drift data, which makes the improved LSTM to be the base classifier for the multi-classifier. As shown in Figure 11, the improved LSTM model is tested in four different datasets. The highest accuracy rate is 97.2% in all tests of dataset 3. Among four kinds of datasets, the highest classification accuracy of 78.0% is obtained on dataset4. Therefore, the results in dataset 4 are adopted in the improved LSTM when comparing with the proposed method ILS below.

#### 4.2.3. Ensemble Multi-Class Classifier

The ILS model adopts the improved LSTM model and the SVM model as the base classifier. The multi-class integration uses the above normalized weighted voting. The dataset under the four setting is the input of the base classifier. The parameter settings of the improved LSTM model and the SVM model are consistent with the parameter selection above.

**Figure 12:**
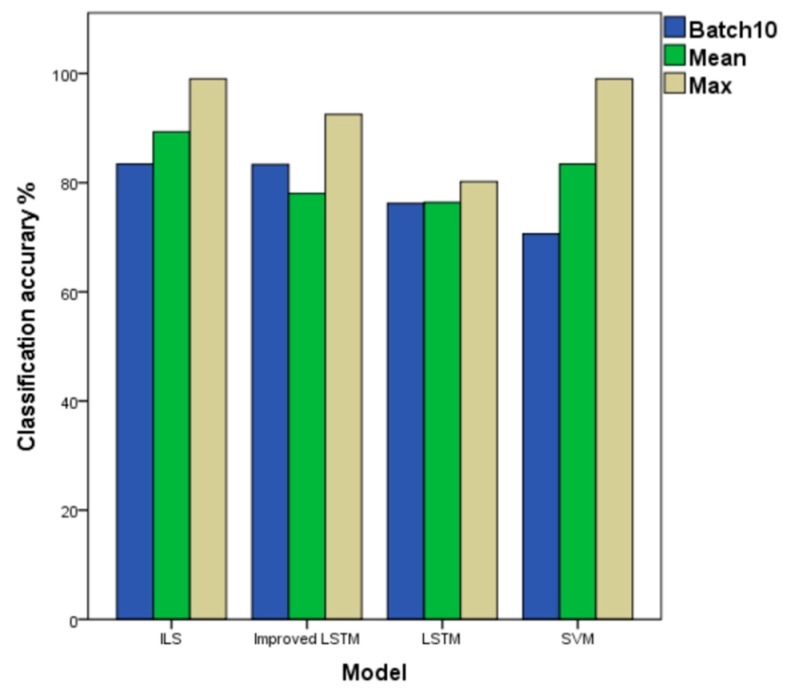
Classification Accuracy of ILS Models and other Models. Green: the highest classification accuracy in every dataset; Blue: the average classification accuracy in every dataset; Brown: the result of batch 10 as a test set in every dataset.

As shown in Table 9, under the SVM, the LSTM and Improved LSTM column represent the ‘previous results’ of the independent classifier, under the ILS column are the result (improved) with the method presented of ensemble classifier. We compare the SVM, LSTM, and improved LSTM models in terms of the highest average accuracy in the four different datasets (Figure 9, Figure 10, Figure 11 and Figure 12 above) to the ILS model in Figure 12, the average accuracy of LSTM, the improved LSTM, SVM, and the ILS model are 76.4, 78.0%, 83.1% and 89.3%, respectively.

The classifier ensemble performs better than the SVM trained at batch points 2, 3, 4, 5, 9, and 10. As mentioned above, this SVM is a very strong baseline and, thus, the ILS performs better than or as well as this SVM is a better result. The ILS model better than the classifier of LSTM at all batches, from batch 3 to batch 10. In the case of batch 9 and batch 10 for testing, the accuracy of the ILS model is 83.4%. In terms of the average accuracy and the accuracy with batch 10 for testing, ILS also performs better. In the highest classification accuracy rate, the ILS model reaches a maximum of 99.0%. Note that, although the Improved ILSM helps to slightly improve the performance of the LSTM trained, it performs worse than some of the batches. 

**Figure 13:**
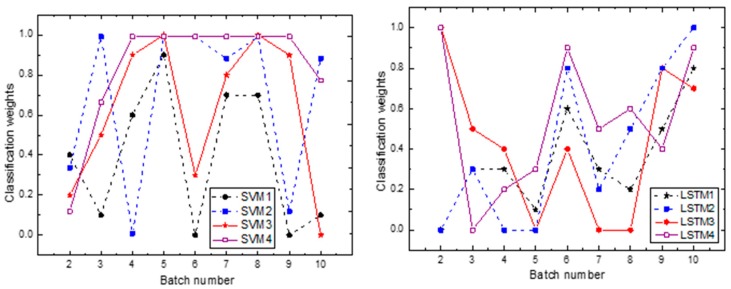
Classifier weights used in the ensembles (SVM1 to SVM4 and, LSTM1 to LSTM4). At every point on the *x*-axis (batch) the corresponding points in the *y*-axis are the weights of the individual classifiers used in the ensemble. Note that these weights max up to 1. Note that the dotted indicates that the input is not a classifier for the correlation analysis dataset and, the solid line indicates that the classifier the inputs the data set for correlation analysis.

We compare the ILS model with the above independent classification, which shows the superiority of the ILS. The model that is presented below is compared with different voting methods of ensemble learning. Figure 13 shows how the classifier weights used in the ILS model change with the batch.

### 4.3. Comparison of Ensemble Voting Methods

In Section 4.2, we compare the integrated multi-classifier ILS with the multi-classifier base classifiers LSTM and SVM. The integrated multi-classifier ILS has better performance than the base classifiers. However, in ensemble learning, the combination of classifiers is equally important. Because in the multi-classifier there are base classifiers that have a negative influence on the predictive capabilities, which affects the predictive ability of ensemble classifiers. There are three ways to vote: majority voting, plurality voting, and weighted voting. For majority voting, the result of the final integration is to select more than half of the votes. That is to say, if more than half of the base learners predict the category c, then the ensemble learner predicts the result as c, otherwise the prediction is rejected. Therefore, in this part, we will compare the proposed classifier combination method with the classical majority voting method and the weighted voting method.

Plurality voting method is predicted to be the mark with the highest number of votes. If multiple marks get the highest number of votes at the same time, a mark is randomly selected from them. We regard the predicted output of $h_{i}$ on sample x as an N-dimensional vector ($h_{i}^{1}(x)$, $h_{i}^{2}(x)$, …, $h_{i}^{N}(x)$), where $h_{i}^{j}(x)$ is the output on the category tag $c_{j}$. The relative plurality voting method indicates:

(22) $$H(x)=c_{\underset{j}{\arg\max}\sum_{i=1}^{T}h_{i}^{j}(x)}$$

The weighted voting method is similar to weighted average, which can be expressed as:

(23) $$H(x)=c_{\underset{j}{\arg\max}\sum_{i=1}^{T}w_{i}h_{i}^{j}(x)}$$

where $w_{i}$ is the weight of $h_{i}$, generally $w_{i}\geq$0, $\sum_{i=1}^{T}w_{i}$ = 1. In this part of the experiment, we compare the plurality method and the weighted voting method with the normalized weighted voting method that is proposed in this paper. The SVM mentioned above and the improved LSTM are selected in the base classifier of the plurality voting method. At the same time, the four datasets that were generated by the same settings above are used as input of the SVM and the improved LSTM. The accuracy of each base classifier is the weight in the weighted voting method (expressed in decimal of weight reuse accuracy).

In Table 10, majority voting is a multi-classifier that uses majority voting as a combined strategy. Plurality voting is the multiple classifier of a combination strategy based on the Plurality voting. In batch 2, 3, 4, 5 as a test sets, the classification accuracy of Majority voting is slightly lower than that of Plurality voting. The main reason is that the lowest accuracy of the base classifier occupies the same weight in the majority voting, which causes a negative effect. When batch 10 is used as the test set, the classification accuracy rate of Plurality voting is 70.7%, and the one of Majority voting is 73.4%.

In the batch 2 and batch 3, the classification accuracy of ILS model and Plurality voting is basically the same, in the average classification accuracy rate, the ILS model reaches 89.2%, the Majority voting and the Plurality voting accounting for 84.2% and 83.9%, respectively. In the ensemble classifiers, if the base classifier with negative influence on the prediction ability has high accuracy, then it has a large negative influence in the voting prediction, and it is easy to make mistakes, which affects the prediction performance of the ensemble classifier. If the base classifiers with the low accuracy in Majority voting occupy the same weight, which also easily affects the predictive performance of the ensemble classifier. In this paper, the normalized weighted vote can be used to cut out some base dividers in the low accuracy. In the predictive stage, the final prediction result is obtained by the weighted allocation of the rest ensemble classifier. Normalized weighting can improve the predictive performance of the ensemble classifier by eliminating the base classifiers with the lowest prediction accuracy in the ensemble classifier.

**Figure 14:**
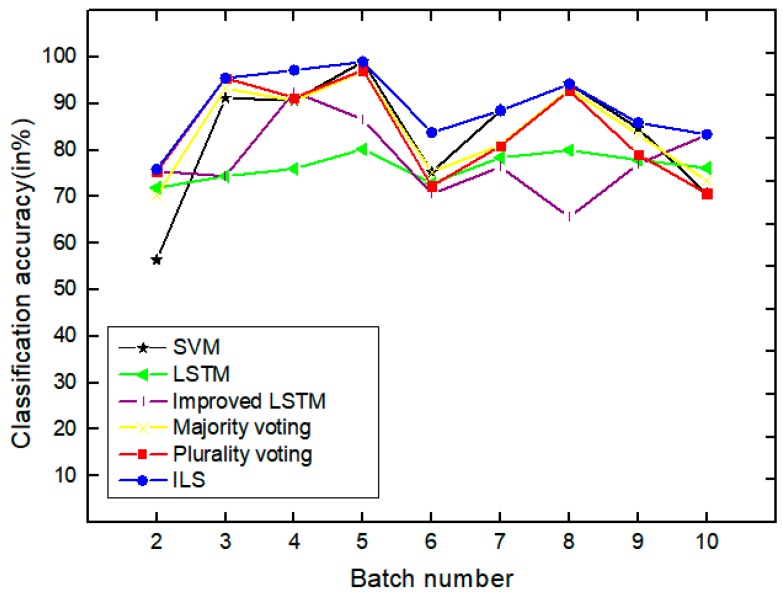
Comparison of all the methods based on achieve classification accuracy for concentration estimation of batch under drift.

As shown in Figure 14, the classifier ensembles are able to perform better than or as well as the classifier trained when tested on most of the most of the batches with significant improvements in accuracy on several batches. Except batch 3, batch 5z, batch 7, and batch 8, ILS has greatly improved the performances and achieved the highest accuracies. The results again turn out to be that the proposed method can effectively promote the classification and process sensor drift by merging drift compensation into the classification task. This result clearly demonstrates the effectiveness of the proposed method for automatic detection and copy with concept drift. 

In terms of cost perspective, the proposed method can train the gas classifiers by the features that were extracted from the datasets. The abstract features extracted by our method can cope with the complex data and non-linear changes, so our method is not only robust, but also universal to the gas sensor drift. The cost of this method is relatively low, because it only requires appropriate marker data without an additional reference gas. The ILS model is composed of the LSTM and SVM classifiers. When compared with SVM, LSTM has a higher time complexity. However, the proposed model does not increase the time complexity. The LSTM model corresponds to four sets of parameters, including input gate, forget gate, output gate, and candidate state. In the LSTM, the parameters can be simplified to two matrices, U and V, which can map the input and output, respectively. The dimension of U is the hidden * input, and the dimension of V is hidden * hidden. Therefore, the network is learning these two matrices, so the total of the LSTM is $4(nm+n^{2}+n)$, where n is hidden_size, m is input_size. The amount of average time consumed by each algorithm is given in Table 11 in order to compare algorithms based on time complexity. 

## 5. Conclusions

The supervised learning algorithm can effectively manage and compensate for the sensor drift. In this study, we propose a multi-classifier integration supervised learning method to compensate for drift in gas sensors. The model takes advantage of SVM, whose capacity of few-shot classification and the long-time memory characteristics of LSTM. Besides, the improved loss function eliminates the polarization caused by using SoftMax in LSTM model. Additionally, it combines SVM with the improved LSTM. Through the normalized weighted voting strategy, the base classifier with the lowest accuracy of the classifier is removed in every voting process to make the proposed model ILS adapt to the sensor drift, which effectively improves the performance of the sensor drift classifier. The model does not make any assumptions about the nature of the drift, so that the model has a better generalization ability. In addition, the used data are collected over a long period of time and it has drift characteristics, which is a relatively comprehensive dataset for exploration. On datasets with four kinds of setting, we conduct a correlation analysis and make it clear that better the approximation results could be obtained with the increased hypothesis space. When compared with SVM, LSTM, and the improved LSTM model, the proposed method achieves highest accuracy 99.0% and average accuracy 83.4%.

Our model has achieved the good experimental results on the current dataset. However, the supervised learning requires huge manpower and resources to compensate the sensor drift in dynamic labeling and training data. Besides, the classifier model has a longer training time than a Random Forest. In the future, we will further investigate the classification of unlabeled data from the sensor drift in a semi-supervised manner and attempt to optimize the model [31].

## Figures and Tables

**Table 1:** Sensors Information in the Sensor Array.

Sensor Type	Number of Units	Target Gases
TGS2600	4	Hydrogen, carbon, monoxide
TGS2602	4	Ammonia, H_2_S, volatile organic compounds (VOC)
TGS2610	4	Propane
TGS2620	4	Carbon monoxide, combustible gases, VOC

**Table 2:** Placement Order of Extracted Features in the Feature Vector.

1. ΔR_S1	9. ΔR_S2	17. ΔR_S3	25. ΔR_S4	…	121. ΔR_S16
2. ‖ΔR‖_S1	10. ‖ΔR‖_S2	18. ‖ΔR‖_S3	26. ‖ΔR‖_S4	…	122. ‖ΔR‖_S16
3. $ema_{0.001}I\text{\_S}1$	11. $ema_{0.001}I\text{\_S}2$	19. $ema_{0.001}I\text{\_S}3$	27. $ema_{0.001}I\text{\_S}4$	…	123. $ema_{0.001}I\text{\_S}16$
4. $ema_{0.01}I\text{\_S}1$	12. $ema_{0.01}I\text{\_S}2$	20. $ema_{0.01}I\text{\_S}3$	28. $ema_{0.01}I\text{\_S}4$	…	124. $ema_{0.01}I\text{\_S}16$
5. $ema_{0.1}I\text{\_S}1$	13. $ema_{0.1}I\text{\_S}2$	21. $ema_{0.1}I\text{\_S}3$	29. $ema_{0.1}I\text{\_S}4$	…	125. $ema_{0.1}I\text{\_S}16$
6. $ema_{0.001}D\text{\_S}1$	14. $ema_{0.001}D\text{\_S}2$	22. $ema_{0.001}D\text{\_S}3$	30. $ema_{0.001}D\text{\_S}4$	…	126. $ema_{0.001}D\text{\_S}16$
7. $ema_{0.01}D\text{\_S}1$	15. $ema_{0.01}D\text{\_S}2$	23. $ema_{0.01}D\text{\_S}3$	31. $ema_{0.01}D\text{\_S}4$	…	127. $ema_{0.01}D\text{\_S}16$
8. $ema_{0.1}D\text{\_S}1$	16. $ema_{0.1}D\text{\_S}2$	24. $ema_{0.1}D\text{\_S}3$	32. $ema_{0.1}D\text{\_S}4$	…	128. $ema_{0.1}D\text{\_S}16$

Where, ΔR_Si and ‖ΔR‖_Si are the *R* and the normalized *R* features, respectively. $ema_{0.001}I\text{\_Si}$, $ema_{0.01}I\text{\_Si}$, and $ema_{0.1}I\text{\_Si}$, are the rising transient portion of the sensor response for a 0.001, 0.01, and 0.1, respectively, $ema_{0.001}D\text{\_Si}$, $ema_{0.01}D\text{\_Si}$, and $ema_{0.1}D\text{\_Si}$, are the decaying transient portion of the sensor response for a 0.001, 0.01, 0.1, respectively. The index i=1,i∈(1,2,…,16) represents the number of the sensor, thus forming 128-dimensional feature vector.

**Table 3:** Correlation analysis processing the partial results.

1. ΔR_S1	9. ΔR_S2	17. ΔR_S3	25. ΔR_S4	33. ΔR_S5	41. null
2. ‖ΔR‖_S1	10. null	18. ‖ΔR‖_S3	26. ‖ΔR‖_S4	34. null	42. ‖ΔR‖_S6
3. $ema_{0.001}I\text{\_S}1$	11. $ema_{0.001}I\text{\_S}2$	19. $ema_{0.001}I\text{\_S}3$	27. null	35. $ema_{0.001}I\text{\_S}5$	43. $ema_{0.001}I\text{\_S}6$
4. $ema_{0.01}I\text{\_S}1$	12. null	20. null	28. $ema_{0.01}I\text{\_S}4$	36. $ema_{0.01}I\text{\_S}5$	44. $ema_{0.01}I\text{\_S}6$
5. $ema_{0.1}I\text{\_S}1$	13. null	21. null	29. $ema_{0.1}I\text{\_S}4$	37. $ema_{0.1}I\text{\_S}5$	45. null
6. null	14. null	22. $ema_{0.001}D\text{\_S}3$	30. $ema_{0.001}D\text{\_S}4$	38. null	46. $ema_{0.001}D\text{\_S}6$
7. $ema_{0.01}D\text{\_S}1$	15. $ema_{0.01}D\text{\_S}2$	23. $ema_{0.01}D\text{\_S}3$	31. null	39. $ema_{0.01}D\text{\_S}5$	47. null
8. $ema_{0.1}D\text{\_S}1$	16. $ema_{0.1}D\text{\_S}2$	24. $ema_{0.1}D\text{\_S}3$	32. $ema_{0.1}D\text{\_S}4$	40. null	48. $ema_{0.1}D\text{\_S}6$

**Table 4:** Basic Data in the Dataset.

Batch ID	Month IDs	Labels	Number of Measurements
batch1	month1, month2	1, 2, 3, 4, 5, 6	445
batch2	month3, month4, month8, month9, month10	1, 2, 3, 4, 5, 6	1244
batch3	month11, month12, month13	1, 2, 3, 4, 5	1586
batch4	month14, month15	1, 2, 3, 4, 5	161
batch5	month16	1, 2, 3, 4, 5	197
batch6	month17, month18, month19, month20	1, 2, 3, 4, 5, 6	2300
batch7	month21	1, 2, 3, 4, 5, 6	3613
batch8	month22, month23	1, 2, 3, 4, 5, 6	294
batch9	month24, month30	1, 2, 3, 4, 5, 6	470
batch10	month36	1, 2, 3, 4, 5, 6	3600

**Table 5:** Sensor Drift Verification.

Train Set Batch	Test Set Batch	Classification Accuracy
1	1	100.0%
1	2	60.3%
1	3	69.9%
1	4	67.7%
1	5	44.7%
1	6	61.0%
1	7	43.2%
1	8	24.8%
1	9	41.7%
1	10	40.7%

**Table 6:** Classification Accuracy of the SVM model under Four Datasets.

Test Set ID	Dataset 1	Dataset 2	Dataset 3	Dataset 4
batch 2	60.3%	60.3%	56.4%	56.4%
batch 3	77.1%	95.5%	84.2%	88.5%
batch 4	84.5%	74.5%	88.8%	90.7%
batch 5	95.4%	99.0%	98.1%	99.0%
batch 6	54.1%	76.0%	60.3%	75.3%
batch 7	82.8%	86.3%	83.9%	88.5%
batch 8	84.0%	92.9%	94.6%	94.2%
batch 9	72.8%	75.7%	83.2%	84.7%
batch 10	40.4%	72.8%	34.5%	70.6%

**Table 7:** Classification Accuracy of the LSTM Model under Four Datasets.

Test Set ID	Dataset 1	Dataset 2	Dataset 3	Dataset 4
batch 2	53.5%	53.5%	71.9%	71.9%
batch 3	79.7%	80.1%	85.7%	74.4%
batch 4	77.7%	73.2%	79.9%	76.0%
batch 5	75.8%	73.6%	72.5%	80.2%
batch 6	66.7%	72.6%	63.3%	72.9%
batch 7	75.9%	74.0%	69.7%	78.4%
batch 8	64.8%	75.3%	56.1%	80.0%
batch 9	78.5%	82.0%	82.6%	77.9%
batch 10	69.1%	78.6%	67.2%	76.2%

**Table 8:** Classification Accuracy of the Improved LSTM Model under Four Datasets.

Test Set ID	Dataset 1	Dataset 2	Dataset 3	Dataset 4
batch 2	72.1%	72.1%	75.5%	75.5%
batch 3	78.0%	61.7%	81.7%	74.4%
batch 4	84.1%	81.4%	97.2%	92.5%
batch 5	83.2%	82.5%	80.1%	86.6%
batch 6	83.8%	75.5%	82.7%	70.6%
batch 7	73.1%	80.1%	65.6%	76.4%
batch 8	54.7%	70.1%	65.8%	65.6%
batch 9	72.5%	76.3%	75.9%	77.0%
batch 10	68.6%	80.3%	72.8%	83.3%

**Table 9:** Classification Accuracy of ILS Models and other Models.

Test Data ID	SVM	LSTM	Improved LSTM	ILS
batch 2	56.4%	71.9%	75.5%	75.9%
batch 3	91.2%	74.4%	74.4%	95.5%
batch 4	90.7%	76.0%	92.5%	97.2%
batch 5	99.0%	80.2%	86.6%	99.0%
batch 6	75.3%	72.9%	70.6%	83.8%
batch 7	88.5%	78.4%	76.4%	88.5%
batch 8	94.2%	80.0%	65.6%	94.2%
batch 9	84.7%	77.9%	77.0%	85.9%
batch 10	70.6%	76.2%	83.3%	83.4%

**Table 10:** Classification Accuracy for different strategies.

Test Data ID	Majority Voting	Plurality Voting	ILS
Batch 2	70.3%	75.3%	75.9%
Batch 3	93.3%	95.5%	95.5%
Batch 4	90.6%	91.3%	97.2%
Batch 5	97.0%	97.2%	99.0%
Batch 6	75.3%	72.3%	83.8%
Batch 7	81.1%	80.9%	88.5%
Batch 8	93.1%	92.8%	94.2%
Batch 9	83.4%	79.1%	85.9%
Batch 10	73.4%	70.7%	83.4%
Mean	84.2%	83.9%	89.3%

**Table 11:** Experimental test time for different prediction models.

Algorithm	SVM	LSTM	Improved LSTM	Majority Voting	Plurality Voting	ILS
Time (s)	0.27	2.55	2.36	5.53	5.72	5.64

## References

[1] Liu Q., Zhou S., Cheng X., Cheng H., Zhang H. Gas Sensor Drift Compensation by an Optimal Linear Transformation. Proceedings of the 2017 3rd International Conference on Big Data Computing and Communications (BIGCOM) IEEE.

[2] Llobet E., Brezmes J., Vilanova X., Sueiras J.E., Correig X. (1997). Qualitative and quantitative analysis of volatile organic compounds using transient and steady-state responses of a thick-film tin oxide gas sensor array. Sens. Actuators B Chem..

[3] Ma Z., Luo G., Qin K., Wang N., Niu W. (2018). Online Sensor Drift Compensation for E-Nose Systems Using Domain Adaptation and Extreme Learning Machine. Sensors.

[4] Bai H., Shi G. (2007). Gas Sensors Based on Conducting Polymers. Sensors.

[5] Lee J.H. (2009). Gas sensors using hierarchical and hollow oxide nanostructures: Overview. Sens. Actuators B Chem..

[6] Ur Rehman A., Bermak A. (2019). Heuristic Random Forests (HRF) for Drift Compensation in Electronic Nose Applications. IEEE Sens. J..

[7] Vergara A., Vembu S., Ayhan T., Ryan M.A., Homer M.L., Huerta R. (2012). Chemical gas sensor drift compensation using classifier ensembles. Sens. Actuators B Chem..

[8] Brahimbelhouari S., Bermak A., Chan P.C.H. Gas identification with microelectronic gas sensor in presence of drift using robust GMM. Proceedings of the IEEE International Conference on Acoustics IEEE.

[9] Liu T., Li D., Chen J., Chen Y., Yang T., Cao J. (2018). Gas-Sensor Drift Counteraction with Adaptive Active Learning for an Electronic Nose. Sensors.

[10] Distante C., Leo M., Siciliano P., Persaud K.C. (2002). On the Study of Feature Extraction Methods for an Electronic Nose. Sens. Actuators B Chem..

[11] Carmela L., Levyb S., Lancetc D. (2003). A feature extraction method for chemical sensors in electronic noses. Sens. Actuators B Chem..

[12] Artursson T., Eklöv T., Lundström I., Mårtensson P., Sjöström M., Holmberg M. (2000). Drift correction for gas sensors using multivariate methods. J. Chemom..

[13] Padilla M., Perera A., Montoliu I., Chaudry A., Persaud K., Marco S. (2010). Drift compensation of gas sensor array data by Orthogonal Signal Correction. Chem. Intell. Lab. Syst..

[14] Liu Q., Hu X., Ye M., Cheng X., Li F. (2015). Gas Recognition under Sensor Drift by Using Deep Learning. Int. J. Intell. Syst..

[15] David E., Netanyahu N.S. (2015). DeepSign: Deep Learning for Automatic Malware Signature Generation and Classification. Int. Jt. Conf. Neural Netw. (IJCNN).

[16] Chen Y., Lin Z., Zhao X., Wang G., Gu Y. (2014). Deep Learning-Based Classification of Hyperspectral Data. IEEE J. Sel. Top. Appl. Earth Obs. Remote Sens..

[17] Marmanis D., Datcu M., Esch T., Stilla U. (2016). Deep Learning Earth Observation Classification Using ImageNet Pretrained Networks. IEEE Geosci. Remote Sens. Lett..

[18] Adhikari S., Saha S. Multiple classifier combination technique for sensor drift compensation using ANN & KNN. Proceedings of the 2014 IEEE International Advance Computing Conference (IACC).

[19] Zhang L., Zhang D. (2015). Domain Adaptation Extreme Learning Machines for Drift Compensation in E-Nose Systems. IEEE Trans. Instrum. Meas..

[20] Liu Q., Li X., Ye M., Ge S.S., Du X. (2014). Drift Compensation for Electronic Nose by Semi-Supervised Domain Adaption. IEEE Sens. J..

[21] Wenwei S., Huichang S. (2005). Sensor drift compensation method based on cyclic neural network. Comput. Eng. Sci..

[22] Wang X., Wu J., Liu C., Yang H., Du Y., Niu W. (2018). Exploring LSTM based recurrent neural network for failure time series prediction. J. Beijing Univ. Aeronaut. Astronaut..

[23] Zhigang J., Yue H., Qi Z. (2018). An emotion analysis model combining deep learning and emsemble learning. J. Harbin Inst. Technol..

[24] Pardo A., Marco S., Samitier J. (1998). Nonlinear inverse dynamic models of gas sensing systems based on chemical sensor arrays for quantitative measurements. IEEE Trans. Instrum. Meas..

[25] Muezzinoglu M.K., Vergara A., Huerta R., Rulkov N., Rabinovich M.I., Selverston A., Abarbanel H.D.I. (2009). Acceleration of chemo-sensory information processing using transient features. Sens. Actuators B Chem..

[26] Xiong J., Liang Q., Wan J., Zhang Q., Chen X., Ma R. (2018). The Order Statistics Correlation Coefficient and PPMCC Fuse Non-Dimension in Fault Diagnosis of Rotating Petrochemical Unit. IEEE Sens. J..

[27] Choi E., Schuetz A., Stewart W.F., Sun J. (2016). Using recurrent neural network models for early detection of heart failure onset. J. Am. Med. Inform. Assoc. JAMIA.

[28] Verma M., Asmita S., Shukla K.K. (2016). A Regularized Ensemble of Classifiers for Sensor Drift Compensation. IEEE Sens. J..

[29] Zhang L., Zhang D., Yin X., Liu Y. (2016). A Novel Semi-Supervised Learning Approach in Artificial Olfaction for E-Nose Application. IEEE Sens. J..

[30] Namrata V., Bouwmans T., Javed S., Narayanamurthy P. (2018). Robust Subspace Learning: Robust PCA, Robust Subspace Tracking, and Robust Subspace Recovery. IEEE Signal Process. Mag..

[31] Schölkopf B., Chapelle O., Zien A. (2013). Semi-Supervised Learning. Handbook on Neural Information Processing.

